# Chromosome Abnormalities and Fertility in Domestic Bovids: A Review

**DOI:** 10.3390/ani11030802

**Published:** 2021-03-12

**Authors:** Alessandra Iannuzzi, Pietro Parma, Leopoldo Iannuzzi

**Affiliations:** 1 Institute for Animal Production System in Mediterranean Environment, National Research Council, 80055 Portici, Italy; alessandra.iannuzzi@cnr.it; 2 Department of Agricultural and Environmental Sciences, University of Milan, 20133 Milan, Italy; pietro.parma@unimi.it

**Keywords:** chromosome abnormality, cattle, river buffalo, sheep, goat, fertility

## Abstract

**Simple Summary:**

In domestic bovids, numerical autosome abnormalities have been rarely reported, as they present abnormal animal phenotypes quickly eliminated by breeders. However, numerical abnormalities involving sex chromosomes and structural (balanced) chromosome anomalies have been more frequently detected because they are most often not phenotypically visible to breeders. For this reason, these chromosome abnormalities, without a cytogenetic control, escape animal selection, with subsequent deleterious effects on fertility, especially in female carriers.

**Abstract:**

After discovering the Robertsonian translocation rob(1;29) in Swedish red cattle and demonstrating its harmful effect on fertility, the cytogenetics applied to domestic animals have been widely expanded in many laboratories in order to find relationships between chromosome abnormalities and their phenotypic effects on animal production. Numerical abnormalities involving autosomes have been rarely reported, as they present abnormal animal phenotypes quickly eliminated by breeders. In contrast, numerical sex chromosome abnormalities and structural chromosome anomalies have been more frequently detected in domestic bovids because they are often not phenotypically visible to breeders. For this reason, these chromosome abnormalities, without a cytogenetic control, escape selection, with subsequent harmful effects on fertility, especially in female carriers. Chromosome abnormalities can also be easily spread through the offspring, especially when using artificial insemination. The advent of chromosome banding and FISH-mapping techniques with specific molecular markers (or chromosome-painting probes) has led to the development of powerful tools for cytogeneticists in their daily work. With these tools, they can identify the chromosomes involved in abnormalities, even when the banding pattern resolution is low (as has been the case in many published papers, especially in the past). Indeed, clinical cytogenetics remains an essential step in the genetic improvement of livestock.

## 1. Introduction

After discovering the Robertsonian translocation rob(1;29) in the Swedish red cattle breed [1], and the demonstration of its harmful effect on fertility [2,3,4], the cytogenetics applied to domestic animals have been widely expanded in many laboratories in order to find relationships between chromosome abnormalities and their phenotypic effects, primarily in terms of fertility.

However, in the years immediately following this discovery, various cytogeneticists published reports on chromosome abnormalities, mostly involving sex chromosomes, underlining the importance of these types of abnormalities, often responsible for sterility, especially in females [5,6,7,8,9,10,11].

Numerical autosome abnormalities have been rarely reported, as they present abnormal animal phenotypes quickly eliminated in early embryo development or by breeders [12]. In contrast, numerical sex chromosome abnormalities and structural (balanced) chromosome anomalies have been more frequently detected in domestic bovids because they are most often not phenotypically visible to breeders (Table 1). For this reason, these chromosome abnormalities, without cytogenetic control, escape selection, with subsequent harmful effects on fertility (and production), especially in female carriers. Chromosome abnormalities can also be easily spread by offspring, especially when using artificial insemination, with adverse economic effects on animal breeding.

The advent of chromosome-banding and FISH-mapping techniques with specific molecular markers (generally BAC clones), reviewed by [13], as well as chromosome painting probes (Zoo-FISH) [14,15], the use of CGH arrays [16], and the availability of standard chromosome nomenclatures [17], have led to the development of powerful tools for cytogeneticists in their daily work. With these tools, they can identify the chromosomes involved in abnormalities and the possible loss or gain of genetic material (especially using CGH arrays). Indeed, clinical cytogenetics remains an essential step in the genetic improvement of livestock.

In this review, we discuss the most crucial chromosome abnormalities (CA) found in domestic bovids (mainly cattle, sheep, goats, and river buffalo) by grouping most of them in tables to synthetize the data. We also suggest possible strategies for a better investigation of CA in animal populations, using efficient and simple banding and molecular techniques to speed up the analyses for the improved selection of reproductive animals.

## 2. Numerical Chromosome Abnormalities

### 2.1. Autosomes

**Figure 1:**
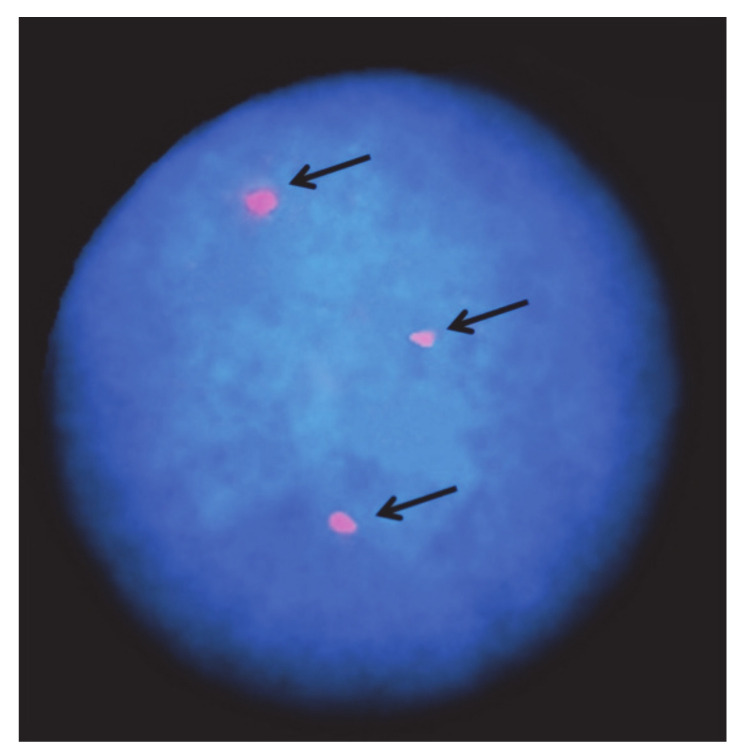
Interphase nucleus of a female cattle calf affected by trisomy 28. Arrows indicate the three FITC signals of the BAC clone containing the conglutinin (CGN1) gene, the official marker of BTA28 (ISCNDB2000, 2001).

Numerical autosome abnormalities have been rarely found in domestic bovids because they are directly eliminated in early embryo development or by breeders when severe anatomical defects occur [12]. Most trisomies reported in cattle involve multiple and heterogeneous defects, especially including those of the muscular-skeletal, cardiovascular, and urogenital systems. Table 2 summarizes the numerical autosomal abnormalities found so far in cattle. Due to the poor banding techniques available in the past, as well as the lack of the use of specific chromosome markers in the FISH-technique in most studies, the accuracy of the chromosome identification can be doubtful. An example is trisomies 22 [18,19] and 28 [20], found in the same animal, when the case was revisited some years after the previous studies, using the same animal slides, chromosome banding, and FISH-mapping technique (Table 2, Figure 1).

Large chromosomes were no longer found to be involved in the autosomal trisomies (see Table 2), probably due to the fetus’s lethal condition, which caused it to die in early embryonic life.

A particular case has been reported in a calf of the Agerolese breed (southern Italy). This animal, unable to stand up and which died a few weeks after birth, was found to be affected by partial trisomy 25 and partial monosomy 11 [39] (Table 2) due to an unbalanced meiosis of the mother cow, which had been affected by a balanced rcp(11;25) and reduced fertility [41]. Two cases of trisomy involving BTA20 and BTA29 have been found using only genomic analyses [30,40]. It should be interesting to compare this approach with cytogenetic analyses, such as chromosome banding and FISH mapping using specific chromosome markers, as recently performed in a case of tandem fusion translocation [42]. A useful approach to detecting numerical chromosome abnormalities using a FISH-mapping technique with two marker chromosomes has been applied to cattle embryos derived from in vitro production (IVP) [43]. These authors observed an increased number of mixoploid cells (diploid and polyploid) compared to in vivo embryos obtained by superovulation (72% of IVP blastocysts versus 25% in vivo). However, the authors maintain that the survival of most calves derived from IVP indicates that a considerable number of these embryos can compensate for the adverse effects of the in vitro procedures [43]. The in vitro aspect is very interesting regarding chromosomal abnormalities, especially in a breeding context. Future breeding might involve in vitro embryo production, subsequent genotyping of the embryo, and selection. In this respect, looking for structural abnormalities will be very important because they will often escape “regular” genomic selection protocols.

### 2.2. Sex Chromosomes

Sex chromosome abnormalities are generally better tolerated by animal species, including the bovids, because one of the X chromosomes genetically is inactivated as gene dosage compensation [44]. However, some genes escape inactivation and cause reproductive disorders involving the abnormal development of internal sex organs [45]. The sex chromosomes of domestic bovids are easily identifiable by both standard chromosome-staining and C-banding techniques. In fact, the X chromosomes of domestic bovids have a different size, shape, and C-banding pattern compared with the autosomes, in particular, (a) BTA-X is submetacentric when all autosomes are acrocentric; (b) BBU-X is the largest acrocentric chromosome, with typically one extensive centromeric C band (and an additional, proximally located C band), compared to all acrocentric autosomes; (c) OAR-X and CHI-X are acrocentric with visible p arms and negative C banding; (d) and BIN-X is submetacentric (as in BTA-X).

The Y chromosome can also be easily detected by both standard chromosome staining (cattle, sheep and goat) or C-banding techniques (river buffalo and zebu). Indeed, the Y chromosome is small and submetacentric in cattle and small and metacentric in both sheep and goat (where the other acrocentric autosomes are all acrocentric). The Y chromosome is acrocentric in both river buffalo and zebu, presenting a positive, distally located C band (C-banding patterns are centromeric in all remaining autosomes). More detailed information about sex chromosome banding is available in [46].

#### 2.2.1. X Trisomy

X trisomy has been rarely found in domestic bovids. The few cases found have only occurred in cattle and river buffalo (Table 3). 

**Figure 2:**
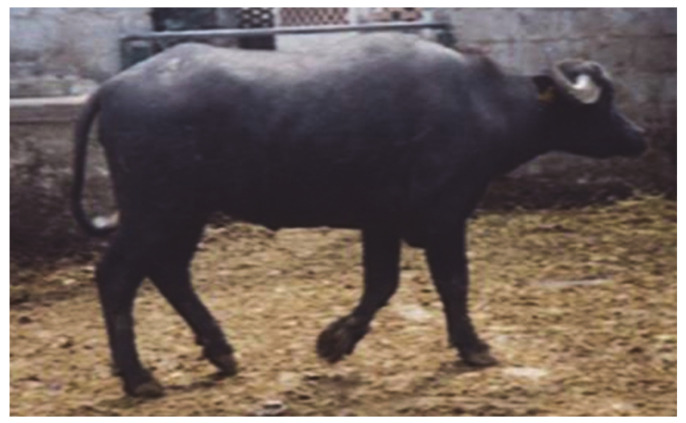
Female river buffalo, five years old, affected by X trisomy (2*n* = 51, XXX). Note the prominent withers (male trait).

Generally, X-trisomic females have a normal body conformation and external genitalia, although a female river buffalo with male traits (prominent withers, tight pelvis, and large horns) has been observed (Figure 2). Carriers are generally affected by infertility (cattle) or sterility (river buffalo) due to damage to the internal sex structures, including ovarian hypoplasia, smaller uterus body, and lack of estrus. As has been established, one of two X chromosomes is randomly inactivated in these females during meiosis as gene-dosage compensation. The same inactivation occurs in X-trisomy cases where one X chromosome is active and the other two are inactivated. Still, abnormalities may result from the presence of three active X chromosomes in early embryonic development, either before X inactivation or due to X-linked genes that escape the inactivation process [56]. In humans, this syndrome is the most common sex chromosome abnormality (1/1000 births, [56]).

#### 2.2.2. X Monosomy

This type of chromosome abnormality is also rare in domestic bovids. Indeed, only a few cases have been recorded so far (Table 4). 

**Figure 3:**
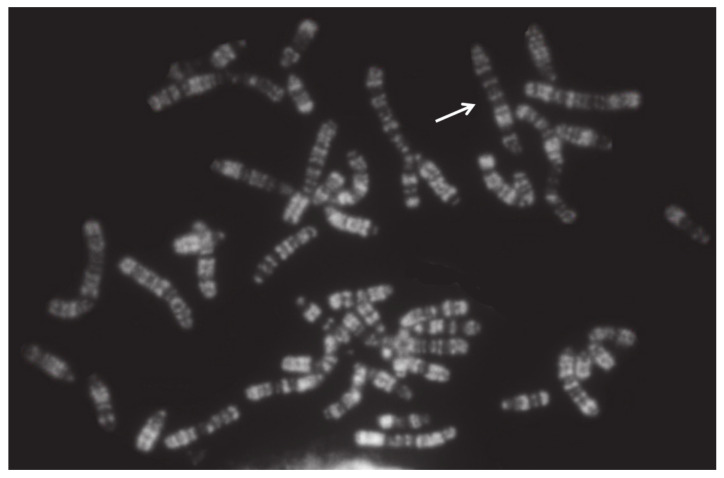
RBA-banding river buffalo metaphase from a female affected by X monosomy (2*n* = 49,X). The only active X chromosome (arrow) was observed in all metaphases. This female was sterile due to damage to her internal sex organs.

Generally, females carrying X monosomy (active X, Figure 3) showed gonadal dysgenesis and sterility [57,58,59,63,68], although in sheep, the effects on the internal sex organs can be less damaging (Table 4), [64,66]. In humans, 1 in 5000 live births is 2*n* = 45,X. In addition, 45,X represents one of the most common chromosome abnormalities identified in spontaneous abortions [56]. Very probably, the same occurs in domestic bovids, complicating the cytogenetic analyses of aborted fetuses. Thus, it is difficult to know the real frequency of this chromosome abnormality in domestic bovids and its fertility effects.

#### 2.2.3. XXY Syndrome

Known in humans as Klinefelter’s syndrome, this abnormality has rarely been found in males of domestic bovids (Table 5). 

**Figure 4:**
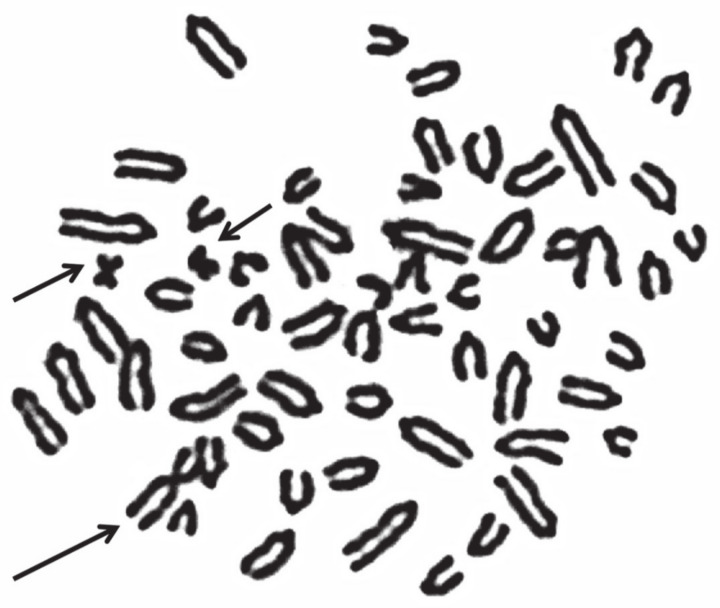
Normal Giemsa-staining metaphase plate of young male cattle for reproduction but promptly eliminated because it was found to be affected by XY/XYY mosaicism. The X chromosome (large arrow) and Y chromosomes (small arrows) are indicated.

Even when two or more X chromosomes are present, the presence of only one Y chromosome is sufficient to induce testes development. This is due to the presence of the SRY gene on the Y chromosome. Carriers are generally affected by testicular hypoplasia, as found also in several cases of mosaicism, XY/XX/XXY, XX/XXY, or XXY/XY (Table 5). An interesting XXY case has been reported in a river buffalo [81]. This male, showing gonadal dysgenesis, presented an unusual karyotype: 2*n* = 50,Y, rob(X;X). A case of mosaicism XY/XYY was found in a young male of the Chianina cattle breed intended for reproduction (Figure 4, Table 5). The animal was promptly eliminated after a karyotype analysis, and it was not possible to further investigate the case.

### 2.3. Sex Reversal Syndrome

This syndrome occurs when male and female phenotypes (or gonadic sex) differ from the expected sex chromosome constitution, as in XX males and XY females. All cases found with this syndrome in domestic bovids are reported in Table 6.

#### 2.3.1. XY Sex Reversal

Bovine XY sex reversal has been observed much more frequently than its counterpart (i.e., XX sex reversal syndrome). Several cases have been reported in this species (Table 6). When the SRY gene sequences were published [103], a test for this syndrome in animals revealed a lack of SRY gene sequences by both PCR and FISH-mapping analysis in such individuals [92,93]. Only two cases of XY sex reversal syndrome have been reported in river buffalo (Table 6). Both females were sterile with severe disruption to their internal sex organs. However, upon investigation by both FISH-mapping and gene-sequence analysis, one individual displayed the SRY gene at its expected location on the Y chromosome with its normal DNA sequence [55]. Similar cases have been reported in sheep [97]. Other authors [104] reported a case of a woman with a 46,XY karyotype and a female phenotype, including histologically normal ovaries. This phenotype, which originated from loss of function due to mutations on the CBX2 gene (human homolog of mouse gene M33), is the only known report of an XY sex reversal with ovary development.

#### 2.3.2. XX Sex Reversal

This syndrome is very rare in domestic animals [105]. Although very rare, XX human males show a variety of clinical manifestations from a normal male phenotype to ambiguous genitalia in newborns. The syndrome is correlated to a translocation of the SRY gene from the Y chromosome to the X chromosome in about 80% of XX sex reversal cases [106,107]. An essential role in this syndrome is played by the chromosome position of the SRY gene in the Y chromosome. When it is located close to the PAR region (as in humans), there are more probabilities for translocations from the Y to X chromosomes during meiotic recombination. In domestic animals, the SRY gene is generally located far from the PAR region [108,109,110], thus explaining its rare occurrence in domestic animals. No documented XX sex reversal related to the SRY gene have been found so far in domestic animals [111,112]. Detailed information on sex reversal syndrome in placental animal species has been reviewed by Parma et al. [113].

### 2.4. XX/XY Mosaicism (Free-Martinism)

This syndrome is the most common sex chromosome abnormality found in domestic bovids in twins of different sexes. In cattle, about 90% of twins of different sexes are free-martin [80,114]. In dairy cattle, the percentage of free-martin twins is higher than that in meat breeds. It varies between 0.5% and 2.0%, with the rate of twinning in dairy breeds between 1% and 4% [115] when the male–female sex ratio is 1:1. Twin pregnancy percentages are also influenced by seasonal effects, reaching the highest levels during springtime and in older dairy cows (6%) [116]. Alterations of internal sex traits seem to be more severe in females than in males, although studies following several free-martin males also reported damage to interior male features [114]. In Italian Friesian cattle, most females with chromosome abnormalities (13%) were free-martin [80]. The presence of XX/XY mosaicism has been found also in bone marrow cattle cells [5].

**Figure 5:**
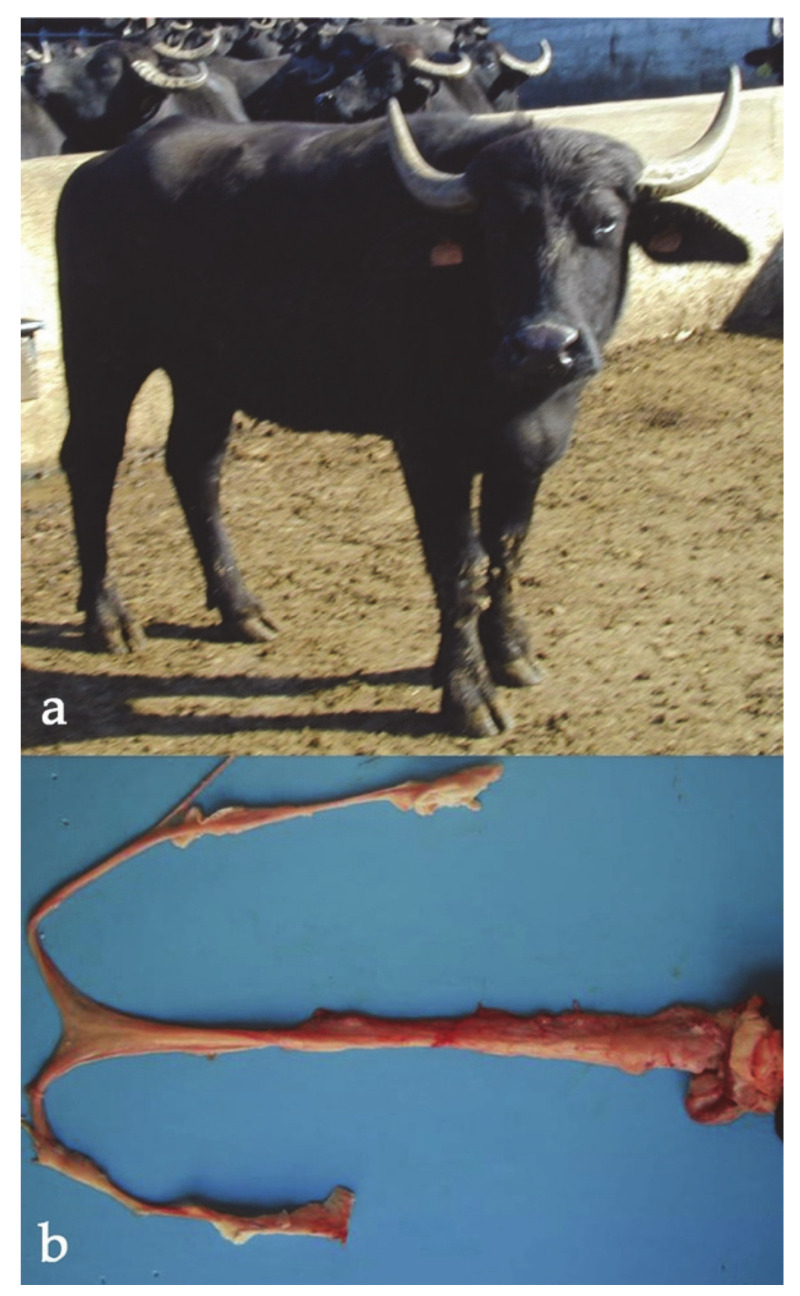
(**a**) River buffalo female showing normal body conformation and external genitalia but found with XX/XY mosaicism (free-martin). Note the atrophic uterine body (**b**).

Free-martin females generally show the typical body conformation and external genitalia. Still, they have pronounced gonadal dysgenesis, varying from a complete lack of internal sex organs (closed vagina) to Mullerian-duct atrophy (Figure 5). Furthermore, several studies reported that damage to the internal sex structures is not correlated with the percentage of male cells in either cattle [116] or river buffalo [117]. Indeed, in both cattle and river buffalo, aberrant internal sex organs were found even in the presence of small percentages of male cells [117]. This is essentially due to three events: (1) placental anastomosis occurring at 20–25 days of embryonic life; (2) sex differentiation occurring later (at 40–45 days) in cattle; and (3) male sex differentiation occurring one week before females [118]. For this reason, the presence of male cells, even in low percentages (and male hormones, in particular AMH), affects the development of internal female sex characteristics [118,119]. For this reason, male free-martins seem to be less prone to abnormal sex anomalies. However, some cases of reduced fertility have been reported in free-martin males [120,121,122,123]. The presence of material belonging to the Y chromosome has also been identified in female subjects with reduced reproductive efficiency [124].

Many free-martin cases are from single births (the other twin dying during early embryonic development). In river buffalo, about 90% of free-martin females were born in single births [55]. This phenomenon is essential because these females generally show normal body conformation and external genitalia, thus escaping breeding selection, unlike in twin births. In the latter case, the breeder knows that the female is probably free-martin and requires a veterinary examination by rectal palpation and cytogenetic or molecular (PCR with specific sex markers) analyses to confirm it.

In sheep and goats, although twins are frequent (but also triplets or quadruplets in some breeds), XX/XY mosaicism correlated to free-martinism occurs at very low frequencies (5−6%) in twins of different sexes, probably because sex differentiation occurs much earlier in sheep (20–25 days after fertilization) than in cattle [125]. Several cases of free-martins have been reported in both sheep [114] and goats [126,127,128], although the frequency of free-martinism is much lower in sheep and goats than in cattle and river buffalo. Sheep and goats carrying XX/XY mosaicism show a pronounced presence of both male and female traits, easily recognizable by breeders [98,129,130].

### 2.5. Diploid-Triploid XX/XXY Mosaicism (Mixoploidy)

This syndrome is very rare in both humans and animals. In domestic bovids, only four cases have been reported of cattle with 2*n* = 60,XX and 3*n* = 90,XXY mosaicism [131]. Generally, the mixoploidy depends on the type of cell in cattle and humans, triploid cells being absent or present in lower percentages in blood lymphocytes and present in higher percentages in fibroblasts or cells of the uterine body or limbs [131,132,133]. In humans, the few 46,XX/69,XXY cases fall into three phenotypic groups: males with testicular development, ovo-testicular disorder of sex development (DSD), or under-virilized male DSD [134]. In cattle, the four cases reported so far showed various phenotypes, including aplasia of the vulva, a rudimentary penis, the presence of ovaries, an empty scrotum, and ovaries with corpus luteum [131].

## 3. Structural Chromosome Abnormalities

### 3.1. Reciprocal Translocations

Reciprocal translocations (rcp) have been found only in cattle and sheep (Table 7).

Rcp are generally balanced, and for this reason, animal carriers show a normal body conformation. Still, they have reduced fertility due to disturbances that occurred during meiosis caused by abnormal (quadrivalent) configurations and erroneous chromosome disjunctions, which can give rise to abnormal embryos that generally die during early embryonic life [138,141,161,162,163]. Without a cytogenetic analysis, these abnormalities escape genetic selection and spread in the offspring, especially when using AI. However, rcp often escape cytogenetic analyses. Most animal cytogenetic labs apply routine cytogenetic analyses with only standard chromosome staining to detect robs, in particular rob(1;29). All cattle autosomes being acrocentric, only when abnormal autosomes are larger and/or shorter than BTA1 and BTA29, respectively, does the lab try to better investigate the case to identify a possible presence of rcp using chromosome-banding techniques and, more recently, chromosome-specific molecular markers (or chromosome-painting probes) by FISH-mapping techniques. For this reason, rcp have been reported with lower frequencies in cattle compared to dicentric robs. A study investigating all rcp found in cattle and correlating them to relative chromosome length concluded that the expected frequency of rcp in cattle is about four times higher than dicentric robs [164]. This estimate is based on two different approaches: (i) a mathematical approach; and (ii) a bioinformatics simulation approach. Both approaches provided similar value and therefore this estimate is believed to be reliable. However, when fertility values, such as (a) the interval between two births, (b) the return to estrus after natural or artificial insemination, and (c) a low number of calves during the reproductive life, appear abnormal, cytogenetic investigations must be done using both chromosome-banding and FISH-mapping techniques [13] to determine the presence, or lack thereof, of chromosome abnormalities like rcp. Generally, only single rcp has been found in bovids, involving only two chromosomes (Table 7). Only rarely has single rcp involved three chromosomes (Table 7) [80,138]. The only case of double rcp involving four chromosomes has been reported by De Schepper et al. [135] (Table 7). Only two rcp involved an autosome and the Y chromosome in an azoospermic bull [145] and a bull negative for testosterone (Table 7) [148].

**Figure 6:**
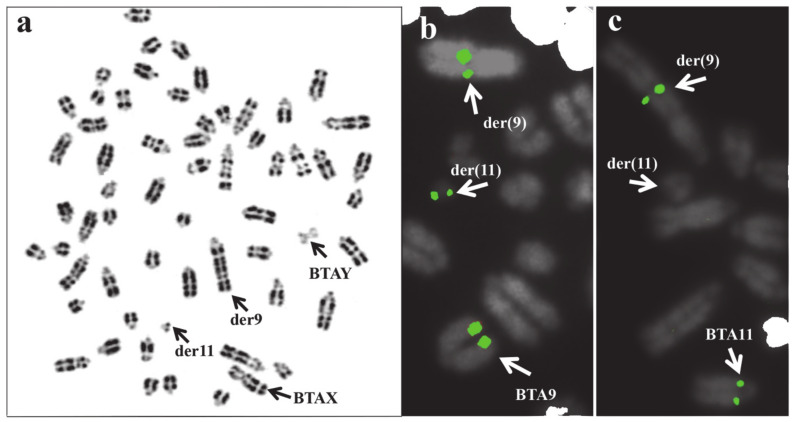
(**a**) Cattle metaphase treated for RBG banding and showing a case of rcp(9;11) (q27;q11) in a young male for reproduction. Arrows indicate the sex chromosomes der(9) and der(11). FISH mapping with two chromosome-specific BAC clones mapping on BTA9 and BTA11 confirmed the chromosomes involved in the rcp (**b**,**c**). Note the presence of FITC signals of a BTA9 marker in BTA9, der(9), and der(11) (**b**), as well as of FITC signals of a BTA11 marker only in BTA11 and der(9), being absent in der(11) (**c**) because the chromosome region was positioned after the break point.

Significant advantages for detecting rcp in domestic bovids (i.e., cattle and sheep) have been derived from improved chromosome-banding and FISH-mapping techniques with specific molecular markers (generally bovine or ovine BAC clones; Figure 6) or chromosome paint probes. Recently, a method using a panel of subtelomeric FISH-probes on a multi-hybridization device, as a means of highlighting the ends of each chromosome, has also been applied to cattle chromosomes to detect structural chromosome abnormalities [153]. However, only two studies extended the analyses using the CGH array to establish possible genetic material losses during chromosome rearrangements (Table 7) [16,149]. At least in these two latter cases, no genetic losses occurred during the rearrangements. Considering that the carriers of rcp are morphologically normal, it is possible to support the hypothesis that the rcp found so far in cattle and sheep are generally balanced.

In humans, the routine uses of genomic investigations allow the study of rcp. Indeed, mapping discordant mate pairs from long-insert, low-pass genome sequencing now permits efficient, cost-effective discovery and nucleotide-level resolution of rearrangement breakpoints, necessary for interpreting the etiology of clinical phenotypes in patients with rearrangements [165]. However, in domestic bovids, because breeders directly eliminate calves showing abnormal phenotypes potentially born from carriers of rcp, it is difficult to study these kinds of mating products.

A rare example has been found in a female calf with partial trisomy 11 and partial monosomy 25, which was unable to stand up and died after a few weeks (Table 2) [39]. The mother of this calf was a carrier of rcp(11;25) (Table 7) [41]. These two latter cases demonstrate that rcp cause reduced fertility by generating unbalanced embryos that die in early embryonic life or a few days after birth.

### 3.2. Robertsonian Translocations (rob)

Centric-fusion translocations are the most common chromosome abnormalities found in cattle. With the exception of rob(1;29), which is monocentric, all remaining robs found in cattle are dicentric (two centromeres; Table 8).

The dicentric translocations reported so far in cattle have generally been found in single cases. Two exceptions are rob(14;20), reported in Simmenthal cattle in both Switzerland and the USA [193,194,195], and rob(26;29), reported in Alpine Grey cattle [139,181,205], where several carriers were found, probably due to the use of AI from bull carriers.

**Figure 7:**
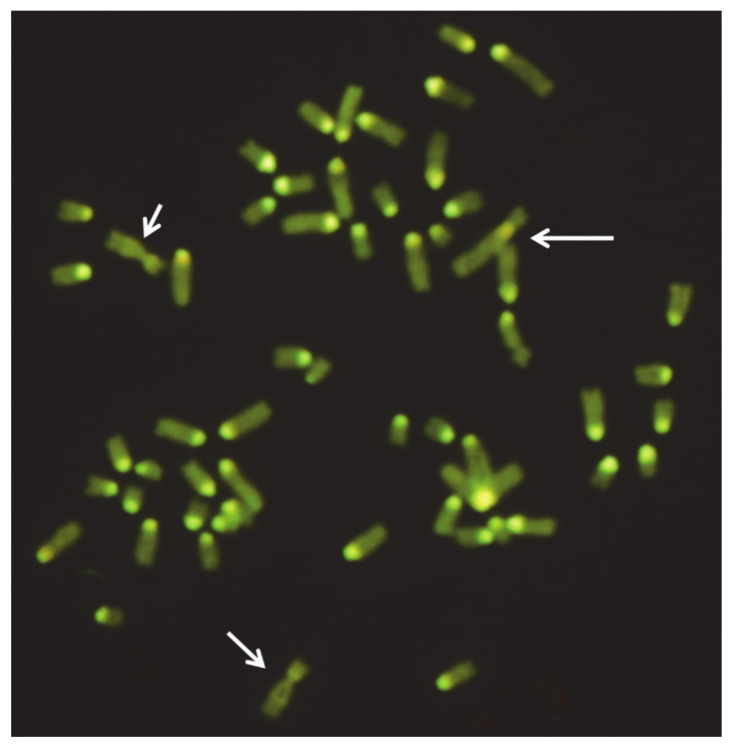
Female cattle metaphase treated for CBA banding in a heterozygous carrier of rob(1;29) (2*n* = 59,XX). Note the single C-band block in the rob(1;29), especially present on the q arms (large arrow). Small arrows indicate X chromosomes.

Generally, dicentric robs disappear after some generations, being unstable due to the presence of two active centromeres and restabilizing to the normal diploid number. In contrast, rob(1;29) is monocentric, showing one (and large) C-banding block particularly present in the q arm (Figure 7). Although this abnormality was discovered a long time ago [1,2], and various studies tried to show the origin of this translocation, only recently and with the use of cytogenetic (high-resolution chromosome banding and FISH-mapping techniques) and genomic (CGH array) analyses, was it possible to establish the origin and evolution of this frequent chromosome abnormality. Indeed, a chromosome segment of about 5 Mb translocated from the proximal region of BTA29 to the proximal region of BTA1, with inversion during the evolution of rob(1;29) [224]. A loss of constitutive heterochromatin (C bands) and of some SAT DNA also was observed on rob(1;29) [225,226].

Rob(1;29), first found in Swedish red cattle [1,2], has been found widely in several breeds (more than 50) [227], mainly in meat breeds. Thus, cytogenetic investigations are particularly focused on meat breeds rather than on dairy cattle breeds, where rob(1;29) has rarely been found, probably because the genetic selection is more strictly applied to dairy breeds. Another hypothesis is that the lower frequency is due to the attempt to reduce the meat breeds’ diploid number from 2*n* = 60 to 2*n* = 58 to gain genetic advantages derived from this new genetic linkage between the two chromosomes. The frequency of this translocation varies among cattle breeds, reaching high values in several breeds, in particular in the Barrosa (Portugal), where the frequency of rob(1;29) carriers has been observed at 70%, of which 53.2% were heterozygous carriers (2*n* = 59) and 16.6% were homozygous (2*n* = 58) carriers [228]. This abnormality reduces fertility in the carriers due to the presence of abnormal trivalent meiotic configurations [2,229] originating in unbalanced gametes that give rise to abnormal embryos that die in early embryonic life. The cow returns to estrus but with some delay compared to the normal interval due to the service’s failure after AI [205]. The reduction in reproductive value in cow rob(1;29) carriers is around 8−9% [80], while in the male carriers it appears to be lower. Indeed, meiotic studies by sperm-FISH in two bulls carrying rob(1;29) revealed a lower percentage (around 2%) of abnormal and unbalanced sperm [230] than those achieved in oocytes of four female carriers of rob(1;29), which showed 21.83% diploid oocytes and 4.06% chromosomally unbalanced sets, with significant variation among carriers. However, these studies should be applied to a larger number of carriers (at least to males) to better establish the real reproductive value of bulls carrying the translocation in terms of unbalanced gametes. Sperm-FISH analyses also should be performed not only on the total sperm fraction but primarily on the motile sperm fraction (i.e., the effective sperm which fertilize the oocytes), as demonstrated in a river buffalo bull sperm carrying a rob(1p;18) translocation [231]. A possible effect of bulls carrying robs(16;20) and (14;20) on the development of bovine oocytes fertilized and matured in vitro was assessed on the basis of embryo yield and blastocyst formation [232]. The study demonstrated that, in bulls carrying the 16;20 and 14;20 translocations, in vitro preimplantation embryo development was reduced (compared to fertilization by a bull with a normal karyotype), probably due to genetically unbalanced spermatozoa [232].

A chromosome-specific marker for rob(1;29) has been found, making it possible to directly detect the presence of this translocation on sperm [233]. This marker, and sperm-FISH with specific chromosome markers, could be particularly useful in males bred for reproduction when no karyotype analyses are applied.

In river buffalo, in addition to the five biarmed pairs originating from centric-fusion translocations during the karyotype evolution [234], three more robs have been found so far as chromosome abnormalities in this species (Table 8). Two of them originated from a complex chromosome mechanism: fission of BBU1 and subsequent centric-fusion translocation between BBU1p and BBU23 in a cow with reduced fertility [207], and later with BBU18 in a very famous Italian bull (named Magnifico) of the Mediterranean Italian breed [208]. Since rob(1p;18) was also found in the bull’s offspring [208], the bull was excluded from reproduction by the Italian buffalo breeder association. Analyses in both total and motile sperm fractions of carrier bulls, by triple-color FISH analysis with a pool of specific BAC probes, revealed that normal sperm were 27% and 69% in the total sperm fraction and motile sperm fraction, respectively [231].

The third case of centric-fusion translocation, rob(X;X), found in river buffalo (Table 8) was reported in a case of an XXY bull with testicular hypoplasia (Table 5) [81].

These studies suggested the necessity of applying cytogenetic investigations in this important species, particularly for all males bred for reproduction and all females with reproductive disturbances, in order to increase the fertility and economic value of river buffalo.

The normal karyotype of sheep (*Ovis aries*, 2*n* = 54) has three biarmed pairs (OAR1, OAR2, and OAR3), which originated from centric-fusion translocations on chromosomes homologous to cattle (and goat, ancestral bovid) 1–3, 2–8, and 5–11, respectively [17]. In addition to these normal biarmed pairs, six centric-fusion translocations, as chromosome abnormalities, were found in sheep, of which five were named t1, t2, t3, t4, and t5, and involving goat-cattle homologous chromosomes 6–24, 9–10, 7–25, 5–8, and 8–22, respectively (Table 8) [209,210,211,212]. More recently, rob(8;11) was found in the Churra da Terra Quente sheep breed (Portugal) [214]. Except for the t4 translocation, which disappeared, and the most recent rob(8;11), found in a single case, the remaining four robs (t1, t2, t3, and t5) remained in New Zealand sheep flocks. Homozygous carriers (2*n* = 48 and 2*n* = 46) were later found in these same sheep flocks [235]. At least for t1, t2, and t3, no particular effects on reproduction seemed to be present in the carriers [236].

Several Robertsonian translocations have also been reported in goats (Table 8). Very probably, some robs, like rob(5;15), rob(6;17), and rob(6;15), reported in Saanen goats, are identical [220,221]. As has generally occurred in other bovids, the translocations were reported in single cases, except for those found in the offspring of males carrying the translocation [217]. The authors performed cytogenetic and genealogical analyses on 205 goats, which were descendants of a sire imported from Switzerland. They reported 29.7% and 4.9% heterozygous and homozygous carriers of rob (5;15), respectively.

### 3.3. Simple Translocation

This chromosome abnormality consists of a chromosome segment region translocated from one chromosome to another. It has been rarely reported. A case of a Y;17 translocation was found in a cattle bull, phenotypically normal (normal reproductive organs and testicular function), but with slight pathospermia (oligozoospermia and asthenozoospermia), However, the portions of the Y chromosome with TDF and AZF were not lost [237]. A case of X-autosome translocation was reported involving almost all of chromosome 23 translocating to the p- arms of the X chromosome of a cow [238]. The same translocation was later found in a bull, which showed malformed spermatozoa [162]. Five cases of 1;8 simple translocation (two males and three females), including a carrier of rob(1;29), were reported by [137].

A case of 2q−;5p+ translocation mosaicism has been reported in a bull, identified by chromosome painting using probes generated by conventional microdissection [239]. Its fertility could not be estimated since the owner culled it before reproduction.

### 3.4. Pericentric Inversion

Few cases of pericentric inversions have been reported in cattle. Popescu [240] found a pericentric inversion involving BTA14 in a female bovine showing reduced fertility. Switonsky [241] found a pericentric inversion involving one of the two X chromosomes in a female with reduced fertility. Iannuzzi et al. [242] found a pericentric inversion in the Y chromosome of 12 male offspring (Podolian breed), of which one had a female-shaped head with reduced horn size, signs of udders, a significantly reduced scrotum, and an atrophic penis. Once slaughtered, an atrophic penis, absence of testis, sign of prostate, and absence of internal female organs were observed. All the remaining carriers of the chromosome abnormality showed normal phenotypes.

De Lorenzi et al. [243] found a possible case of pericentric inversion in the autosomes of a young male cattle. Still, after a detailed FISH-mapping analysis, the authors demonstrated that a centromere repositioning had occurred in BTA17. Subsequent CGH and SNP arrays indicated no loss or gain had occurred in the centromeric region of BTA17 or other BTA17 regions [243].

### 3.5. Tandem Fusion (TAN)

The TANs found so far are centromere–telomere (with two active centromeres as revealed by C-banding techniques) and were rarely found in domestic bovids. Hansen [244] found a case of TAN in the red Holstein breed, while two cases of TAN were found in a male and female of Romanian cattle [95], demonstrating the maternal origin of this abnormality by genealogical investigations. The female carrier of TAN showed a lower non-returned rate and had only two offspring, of which one had a normal karyotype and the other carried the same TAN. The evolution of male carriers was fascinating because the first two analyses revealed a large percentage of mitosis with TAN. Subsequent investigations in four examinations revealed a decreasing number of mitosis with TAN until a total lack of TAN occurred. Indeed, six descendants of this bull showed normal karyotypes [95]. A particular case of TAN (1;16) has been found in a Brown Swiss bull affected by anatomical defects with the simultaneous presence of both TAN(1;16) and trisomy 16 [24]. A case of TAN (4;21) was found in a new-born Holstein-Friesian heifer, which was also XX/XY mosaic (free-martin) [245].

A recent TAN case has been found in a female calf affected by hypospadias, growth retardation, and ventricular septal defects [42]. The TAN involved BTA18 and BTA27 with an accompanying loss of genomic sequences, as demonstrated by chromosome banding, FISH mapping, and genome sequencing [42].

### 3.6. Cytogenetically Detectable Deletions and Duplications

Genetic deletions and duplications have been reported in several studies using genomic approaches and have rarely been reported as chromosome abnormalities. This is probably due to the harmful effects of large genomic losses (deletions) or gains (duplications). These conditions can cause the death of embryos in early embryonic life, especially chromosome deletions. Among the few reported cases of chromosome deletions, only two involved an autosome: the first one in an infertile cow [246] and another one, more recently, in a female calf with several anatomic defects (head asymmetry, relocation of the frontal sinus and eye orbits, hypoplastic thymus without neck part, ductus Botalli, unfinished obliteration in umbilical arteries, and a bilateral series of tooth germs in the temporal region) [247]. In this case, mosaic cells were observed, of which 92% were normal (2*n* = 60, XX) and 8% abnormal (2*n* = 60, XX+ mar) due to the presence of a small marker chromosome showing only the centromere and a proximal part due to the deletion of the remaining material [247].

The remaining cases of deletions involve the X chromosome (generally the inactive and late-replicating X). Indeed, chromosome abnormalities are often found on sex chromosomes because they are more tolerated by the species (for gene inactivation in one of the two Xs) and easily discovered for both shape and C-banding, which are different from the autosomes. A Swiss Holstein bovine, affected by hypotrichosis and oligodontia, was found affected by Xq deletion [248]. A large Xq-arm deletion has been found in a cow carrying rob(1;29) [249]. An interesting case of Xp deletion (2*n* = 60, XX) has been found in a young cow of the Marchigiana breed (central Italy) with normal body conformation and external genitalia [250]. Detailed cytogenetic investigation by both C- and R-banding and FISH-mapping techniques showed that almost all the p arms of the late-replicating (inactive) X chromosome were absent. A CGH-array analysis showed that the deletion involved the Xp arm from the telomere to around 39.5 Mb, referring to the BosTau6 cattle genome assembly. This abnormality deletes about 40 Mb of the X-chromosome sequences, but none of them are programmed to escape from inactivation despite the large number of genes deleted, explaining the normal phenotype of the female. However, this carrier gave rise to a female carrying the same deletion, which later would not remain pregnant after several services and was then eliminated from the farm. The second female carrier gave birth to two calves, both females, of which one was normal and another one carried the same deletion. Later, after several failed services the mother carrier was eliminated from the farm [251]. Both female carries had essentially similar reproductive problems.

Only two cases of chromosome duplications correlating to abnormal phenotypes have been reported in cattle. A possible duplication of a survival motor neuron gene (SMN) has been demonstrated in a calf affected by arthrogryposis (a disease characterized by congenital contractures in the limbs having different origins) using extended-chromosome fiber-FISH [252]. Another chromosome duplication of about 99 Kb has been found in BTA18 using a CGH array on an XY female cattle (SRY positive) affected by a disorder of sex development (DSD), although the authors could not demonstrate its relationship with the phenotype [253].

## 4. Conclusions

As shown in this review, there is a strict relationship between chromosome abnormalities and fertility problems in domestic bovids. In particular, numerical abnormalities have been found very rarely because of their phenotypical visibility, resulting in elimination by breeders. On the other hand, numerical sex chromosome abnormalities often escape selection, as the body conformation and external genitalia are generally normal, but are responsible for sterility in most of cases, including free-martinism, or lower fertility. Structural chromosome abnormalities are usually related to lower fertility compared to normal-karyotyped animals. However, centric-fusion translocations are often present in high percentages in meat breeds, particularly rob(1;29). For this reason, many breeder associations required karyotype analyses for males bred for reproduction, especially for AI, only in meat breeds. This choice is only partially correct because animals belonging to dairy breeds are generally not examined. This could cause reproductive problems in animals, as has occurred in the Italian Friesian breed, where 16.2% of the investigated animals (males and females showing reproductive problems) were found to be carriers of sex chromosome abnormalities, especially of XX/XY mosaicism (see [80]). Finally, only with a good collaboration between breeders, veterinary doctors, and cytogeneticists, as well as between different labs that use genomic and/or cytogenetic approaches, is it possible to correctly investigate the presence of chromosome abnormalities and their effects on fertility in domestic animals in order to better select reproductive animals to improve both their genetic and economic value.

## Figures and Tables

**Table 1:** Schematic representation of the chromosome abnormalities in domestic bovids.

Chromosome Abnormalities		
Numerical		Structural
Autosomes	Sex Chromosomes	
Very rare (the animal body conformation being abnormal; these abnormalities are eliminated directly by the breeders)	More tolerated by the species but almost all related to sterility or low fertility, especially in the females Generally not visible in the carriers (normal body conformation and external genitalia)	Deviation from the normal chromosome shape or gene order Very important for the (a) high percentage of carriers (i.e., cattle rob1;29); (b) normal body conformation; (c) because they escape the normal breeding selection They can be balanced (translocations and inversions) or unbalanced (deletions, insertions, and duplications)

**Table 2:** Autosomal trisomies in cattle.

Chromosome Involved	Phenotype	References
Large Autosome	Male calf with extreme brachygnathia inferior	[21]
12	Anatomical defect, lethal	[22,23]
16 (TAN,1;16)	Anatomical defects	[24]
18 (?)	Anatomical defects	[25]
19	Anatomical defects (BI)	[26]
20	Sterile cow	[27]
	Malformed calf, absence of external genitalia	[28]
	Malformed fetus, cranial defects	[29]
	Fetus with pulmonary hypoplasia and anasarca syndrome (genomic analysis)	[30]
21 (?)	Anatomical defects	[31]
21	Newborn Hereford with a cleft palate, hydrocephalus, a cardiac interventricular septal defect, and arthrogryposis	[32]
22	Anatomical defects (no lethality) [33] Multiple malformations, including hypoplasia of palpebral fissures, cleft palate, kyphoscoliosis, and arthrogryposis	[32,33,34]
21 and 27	Fetuses	[35,36]
22 ^1^	Anatomical defects	[18]
	Anatomical defects	[19]
24	Malformed heifer (slight prognathia, heart defects, slow growth rate)	[37]
26	Sterility, growth retardation	[38]
25 +;11−	Anatomical defects	[39]
28 ^1^	Anatomical defects	[20]
29	Malformed female calf showing dwarfism with severe facial anomalies (genomic analysis)	[40]

^1^ Same animal. ? means uncertain chromosome involved.

**Table 3:** X-trisomy in domestic bovids.

Species	Phenotype	Reference
Cattle	Meiotic disturbances, familiar disposition, infertility	[47]
	Infertility	[48]
	Infertility	[22]
	Infertility	[49]
	Continuous estrus	[50]
	Infertility	[51]
	Infertility, 2 cases	[52]
R. Buffalo	Sterile (damages to internal sex structures)	[53]
	Sterile (damages to internal sex structures)	[54]
	Sterile (damages to internal sex structures), male traits	[55]

**Table 4:** X-monosomy in domestic bovids.

Species	Phenotype	Reference
Cattle	Gonadal disgenesis (sterility)	[57]
	Gonadal disgenesis (sterility)	[58]
	Body smaller in size, the uterus and uterine tubes appeared immature and inactive.	[59]
	Infertile heifer (XY/X0/Y-isochromosome)	[60]
R. Buffalo	Gonadal disgenesis (sterility)	[61]
	Gonadal disgenesis (sterility)	[62]
	Gonadal disgenesis (sterility)	[63]
Sheep	Normal phenotype and external genitalia, no nursing of offspring	[64]
	Gonadal dygenesis in the X0/XX karyotype	[65]
	Dizygotic sheep twins with internal sex damages and mammary gland development very limited	[66]
Goat	Gonadal dysgenesis (XO/XX/XXX mixoploidy)	[67]

**Table 5:** XXY-syndrome in domestic bovids.

Species	Phenotype	References
Cattle	Testicular hypoplasia in a mosaicism case XY/XX/XXY	[69]
	Testicular hypoplasia	[70]
	Testicular hypoplasia	[22]
	Intersexuality in a mosaicism case XX/XXY	[71]
	Bilateral testicular hypoplasia	[72]
	Testicular hypoplasia	[11]
	Testicular hypoplasia in a mosaicism case XX/XYY	[73]
	Masculinization effects in a mosaicism case XX/XXY	[70]
	Testicular hypoplasia	[74]
	Testicular hypoplasia (XXY + rob(1;29))	[75]
	2 cases (testicular hypoplasia with degradation of seminiferous tubules in one examined case)	[76]
	Azospermic bull	[77]
	Testicular hypoplasia in a bull with mosaicism (XY/XYY)	[78]
	Testicular hypoplasia	[79]
	Testicular hypoplasia	[80]
	Testicular hypoplasia in 3 cases	[52]
	Young male excluded for reproduction being mosaic for XY/XYY	Present Study
R. Buffalo	Testicular hypoplasia in a case of *2n* = 50,Y, rob(X;X)	[81]
Sheep	2 cases in rams showing hypoplastic testis	[82]
	Ram with no particular phenotypic effects (XX/XYY mosaicism)	[83]
Goat	Testicular hypoplasia in a case of XXY/XY mosaicism	[84]
	XX/XXY fertile buck	[85]

**Table 6:** Cases with sex reversal syndrome in domestic bovids.

Species	Sex Chrom.	Phenotype/Effects on Fertility	Reference
Cattle	XY	Female (2) with reproductive defects	[86]
	XY	Female with internal sex anatomical defects and no estrus	[87]
	XY	Female with no estrus and streak gonads	[88]
	XY	Female with hypoplastic ovaries	[89]
	XY	Single birth female with normal internal sex adducts but feeble estrus	[27]
	XY	Female normal gonads and genital development with AMGY and ZFY genes present (no SRY determination)	[90]
	XY	Female with hypoplastic gonads (the right one resembled an ovary and the left one an undeveloped testis)	[91]
	XY	Females (3) with no estrus and abnormal Y (Yp-iso)	[92,93]
	XX	Male with both testis and ovotestis development	[94]
	XX	Male XX + rob(1;29) apparently with the normal reproductive parameters but eliminated for rob(1;29)	[95]
R. buffalo	XY	Females (2) sterile with abnormal internal sex adducts (one case with SRY-positive)	[55,96]
Sheep	XY	Sterile ewe with streak gonads, SRY+	[97]
	XY	Ewe with a longer ano-vulvar distance, enlarged clitoris, two testes-like structures at the inguinal level	[98]
Goat	XX	Testicular biosynthesis of testosterone	[99]
	XX	Males intersex, SRY-, Polled Intersex Syndrome (PIS)	[100,101,102]

**Table 7:** Reciprocal translocations (rcp) found in cattle and sheep, with the chromosomes involved, phenotypic effects (when available), and author reference.

Species	Rcp/Chrom. Involved	Phenotype	Reference
Cattle	double rcp(2q−;20q +, 8q-;27q +)	reduced fertility	[135]
	rcp(8;15) (q21;q24)	reduced fertility	[136]
	rcp(1;8) (q44:q16)	2 males and 3 females, reduced fertility	[137]
	rcp(1;8;9) (q43;q13;q26)	subfertile bull subfertile bulls (*n* = 3)	[138,139]
	rcp(8;13) (q11;q24)	azoospemic bull	[140]
	rcp(20;24) (q17;q25)	subfertile bull	[141]
	rcp(X;1) (42;13)	normal female calf with mosaicism XX/XY	[142]
	rcp(12;17) (q22;q14)	subfertile bull	[143]
	rcp(1;5) (q21;q35)	azoospermic bull and its dam (reduced fertility)	[144]
	rcp(Y;9) (q12.3;q2.1)	azoospermic bull	[145]
	rcp(11;21) (q28;q12)	bull, no libido, rare spermatozoa	[146]
	rcp(9;11) (q27;q11)	male addressed to reproduction	[147]
	rcp(2;4) (q45;q34)	bull (post mortem SC-analysis)	[148]
	rcp(4;7) (q14;q28)	bull, balanced, cyto-genomic analysis (CGH-arrays)	[149]
	rcp(Y;21) (p11;q11)	bull testosterone negative	[150]
	rcp(11;25) (q24;q11)	cow with reduced fertility	[41]
	rcp(13;26)	cow with reduced fertility	[151]
	rcp(5;6) (q13;q34)	bull, balanced, cyto-genomic analysis (CGH-arrays)	[16]
	rcp(13;26) (q24;q11)	dam and calf, balanced	[152]
	rcp(12;23)	two subfertile bulls	[153]
Sheep	rcp(1p;19q)	low fertility	[154]
	rcp(13;20) (q12;q22)	low fertility	[155]
	rcp(2q;3q)	low fertility	[156,157]
	rcp(2p−;3q +)	low fertility	[80,158]
	rcp(4q;12q) (q13;q25)	low fertility	[159]
	rcp(18;23) (q14;q26)	low fertility	[160]
	rcp(13;20) (q12;q22)	poor fertility	[155]

**Table 8:** Dicentric Robertsonian translocations reported in cattle, river buffalo, sheep, and goat.

Species	Rob/Chrom.		Breed/Country	Reference
Cattle	1	4	Czech Republic	[166]
	-	7	Not reported	[167]
	-		Blond D’Aquitaine, France	[80]
	-	21	Friesian	[168]
	-	22	Czech Republic	[166]
	-	23	Czech Republic	[166]
	-	25	Blonde d’Aquitaine, N.Z. Piebald cattle Germany	[169,170]
	-	26	Friesian, Japan	[171]
	-	27	British Friesian	[172]
	-	28	Czech Republic	[166]
	2	4	Friesian, England	[173]
	-	8	Friesian, England	[167]
	-	27	Not reported	[167]
	-	28	Vietnamese cattle	[174]
	3	4	Limousine, France	[175]
	-	12	Blond D’Aquitaine, France	[80]
	-	16	Montbéliarde, France	[176]
	-	27	Black spotted, Romania	[95]
	4	4	Czech Republic	[167]
	-	8	Chianina, Italy	[177]
	-	10	Blonde d’Aquitaine, France	[178]
	5	18	Simmenthal, Hungary	[179]
	-	21	Japanese Black, Japan	[167]
	-	22	Polish Red White, Poland	[180]
	-	23	Brown, Romania	[95]
	6	8	Chianina, Italy	[177,181]
	-	28	Czech Republic	[166]
	7	21	Japanese Black Cattle, Japan	[182,183]
	8	9	Brown Swiss, Switzerland	[167]
	-	23	Ukrainian Grey	[167]
	9	23	Blonde d’Aquitaine, France	[184]
	10	15	Pitangueiras, Spain	[185]
	11	16	Simmenthal, Hungary	[186]
	-	21	Brown, Romania	[95]
	-	22	Czech Republic	[167]
	12	12	Simmenthal, Germany	[167]
	-	15	Friesian, Argentina	[167]
	13	14	Friesian, Slovakia	[187]
	-	19	Marchigiana, Italy	[188]
	-	21	Friesian, Hungary	[189]
	-	24	Red &White, Poland. Not reported	[80,187,190]
	14	17	Marchigiana, Italy	[191,192]
	-	19	Braunvieh, Switzerland	[167]
	-	20	Simmenthal, Switzerland, USA. Spotted, Romania	[95,193,194,195]
	-	21	Simmental, Hungary	[167]
	-	24	Podolian, Italy	[196]
	-	28	Friesian, USA	[197]
	15	25	Barrosã, Portugal	[198]
	16	18	Barrosã, Portugal	[199]
	-	19	Marchigiana, Italy	[167]
	-	20	Simmenthal, Czeck Rep.	[200,201]
	-	21	RedPied, Czeck Rep.	[167]
	19	21	Friesian, France	[202]
	20	20	Simmenthal, Germany	[167]
	21	27	Blonde d’Aquitaine, France	[203]
	21	23	Maremmana, Italy	[204]
	-	29	Blonde d’Aquitaine, France	[80]
	24	27	Friesian, Egypt	[167]
	25	27	Alpine Grey, Italy	[139]
	26	29	Alpine Grey, Italy	[139,181,205]
	27	29	Guernsey, Canada	[206]
**R. buffalo**	1p	23	Ital. Mediterranean, Italy	[207]
	1p	18	Ital. Mediterranean, Italy	[208]
	X	X	Murrah, India	[81]
**Sheep**	6	24	(t1) New Zeland Romney, NZ	[209,210]
	9	10	(t2) New Zeland Romney, NZ	[210,211]
	7	25	(t3) New Zeland Romney, New Zeland	[210,211]
	5	8	(t4) New Zeland Romney, New Zeland	[212]
	8	22	(t5) New Zeland Romney, New Zeland	[212]
	1	20	Undefined Race, Germany	[213]
	8	11	Churra da Terra Quente, Portugal	[214]
**Goat**	2	13	Undefined Race, France	[215]
	3	7	-	[161]
	5	15	Saanen, Scotland. Saanen, Brazil	[216,217]
	6	17	Saanen, Switzerland. Saanen, Germany	[218,219]
	6	15	Saanen, Italy. Saanen, France. Saanen, Brazil	[220,221,222]
	10	12	Malaguena, Spain	[223]

## Data Availability

Data sharing is not applicable to this article as no new data were created or analyzed in this study.

## Abbreviations

BTA	*Bos taurus* chromosome, 2*n* = 60
BBU	*Bubalus bubalis* chromosome, 2*n* = 50
OAR	*Ovis aries* chromosome, 2*n* = 54
CHI	*Capra hircus* chromosome, 2*n* = 60
BIN	*Bos indicus* chromosome, 2*n* = 60
FISH	fluorescence in situ hybridization
Fiber-FISH	extended chromatin fiber-FISH
CGH-array	comparative genomic hybridization array
DSD	disorder sex development
